# Identification of monocyte-associated genes as predictive biomarkers of heart failure after acute myocardial infarction

**DOI:** 10.1186/s12920-021-00890-6

**Published:** 2021-02-09

**Authors:** Qixin Chen, Qijin Yin, Junxian Song, Chuanfen Liu, Hong Chen, Sufang Li

**Affiliations:** 1 Department of Cardiology, Beijing Key Laboratory of Early Prediction and Intervention of Acute Myocardial Infarction, Center for Cardiovascular Translational Research, Peking University People’s Hospital, No 11. Xizhimen South Street, Xicheng District, Beijing, 100044 China; 2 Ministry of Education Key Laboratory of Bioinformatics, Research Department of Bioinformatics at the Beijing National Research Center for Information Science and Technology, Center for Synthetic and Systems Biology, Department of Automation, Tsinghua University, Beijing, 100084 China

**Keywords:** Heart failure, Acute myocardial infarction, Biomarker, Systems biology, Monocyte

## Abstract

**Background:**

Acute myocardial infarction (AMI) is a major contributor of heart failure (HF). Peripheral blood mononuclear cells (PBMCs), mainly monocytes, are the essential initiators of AMI-induced HF. The powerful biomarkers for early identification of AMI patients at risk of HF remain elusive. We aimed to identify monocyte-related critical genes as predictive biomarkers for post-AMI HF.

**Methods:**

We performed weighted gene co-expression network analysis (WGCNA) on transcriptomics of PBMCs from AMI patients who developed HF or did not. Functional enrichment analysis of genes in significant modules was performed via Metascape. Then we obtained the single-cell RNA-sequencing data of recruited monocytes/macrophages from AMI and control mice using the Scanpy and screened 381 differentially expressed genes (DEGs) between the two groups. We validated the expression changes of the 25 genes in cardiac macrophages from AMI mice based on bulk RNA-sequencing data and PBMCs data mentioned above.

**Results:**

In our study, the results of WGCNA showed that two modules containing 827 hub genes were most significantly associated with post-AMI HF, which mainly participated in cell migration, inflammation, immunity, and apoptosis. There were 25 common genes between DEGs and hub genes, showing close relationship with inflammation and collagen metabolism. CUX1, CTSD and ADD3 exhibited consistent changes in three independent studies. Receiver operating characteristic curve analysis showed that each of the three genes had excellent performance in recognizing post-AMI HF patients.

**Conclusion:**

Our findings provided a set of three monocyte-related biomarkers for the early prediction of HF development after AMI as well as potential therapeutic targets of post-AMI HF.

## Background

Heart failure (HF) is a growing global health problem affecting approximately 26 million people worldwide. It is estimated that more than 40 million people will develop this condition by 2030 [1]. HF has become a major cause of cardiovascular morbidity and mortality, which brought serious financial burden to both developed and developing countries [2, 3]. Despite sufficient improvements in prevention and therapies, HF patients still present high hospitalization rates and poor survival rates [4, 5]. Among different pathogenic factors, ST-segment-elevation myocardial infarction (STEMI), as the most common and severe type of acute myocardial infarction (AMI), is the major contributor of HF [6]. Thus, early recognition of AMI patients at risk of developing HF is an effective strategy for the reduction of HF incidence. Identifying early biomarkers associated with post-AMI HF may be helpful to resolve this issue. Though several biomarkers were proved to be related to HF triggered by AMI, such as natriuretic peptides (NPs), Galectin-3, soluble suppression of tumorigenicity-2 (sST2), growth/differential factor-15 (GDF-15) [7, 8], powerful biomarkers for early prediction of post-AMI HF remain elusive.

Cardiac remodeling, a major pathological change of HF, is characterized by cells apoptosis and necrosis, inflammation and immune cells activation, cardiomyocyte hypertrophy and myocardial interstitial fibrosis [9]. Increasing evidence indicated that ~ 25% of survivors after STEMI developed HF due to cardiac remodeling [10–13]. PBMCs, mainly monocytes, are essential regulators of cardiac remodeling after AMI [9]. Monocytes rapidly move from bone marrow and spleen to blood and infiltrate the infarct zone and participate in the inflammatory response hours after myocardial injury [14, 15]. Hence, genes involved in recruited monocytes/macrophages-triggered cardiac remodeling may serve as promising biomarkers for the early identification of AMI patients at risk of HF development.

High-throughput microarray and transcriptome sequencing (such as bulk RNA- or single-cell RNA-sequencing) permit us to perform a quick and comprehensive detection over the gene expression profiling, which are unbiased methods for screening disease-specific biomarkers. Based on the transcriptomics data, several studies have identified potential biomarkers for prediction of HF following AMI via differentially expressed genes (DEGs) analysis [16–18], whereas which may result in the omission of some key genes related to disease. Weighted gene co-expression network analysis (WGCNA), as a bioinformatics application, can provide rich information based on calculating the pair-wise correlations between gene expression profiles [19]. WGCNA is thus increasingly used to identify key genes (termed hub genes) associated with specific disease or clinical trait [20–24].

Herein, we aimed to investigate monocyte-related critical genes as predictive biomarkers for HF development following AMI based on WGCNA. By using integrated analyses of gene expression profiles from microarray (samples: PBMCs from AMI patients), single-cell RNA-sequencing (scRNA-seq, samples: recruited monocytes/macrophages from AMI mice) and bulk RNA-sequencing (bulk RNA-seq, samples: cardiac macrophages from AMI mice), we identified 3 key genes CUX1, CTSD, and ADD3 as potential biomarkers for early recognizing AMI patients at risk of developing HF.

## Methods

### Data collection and processing

**Fig. 1:**
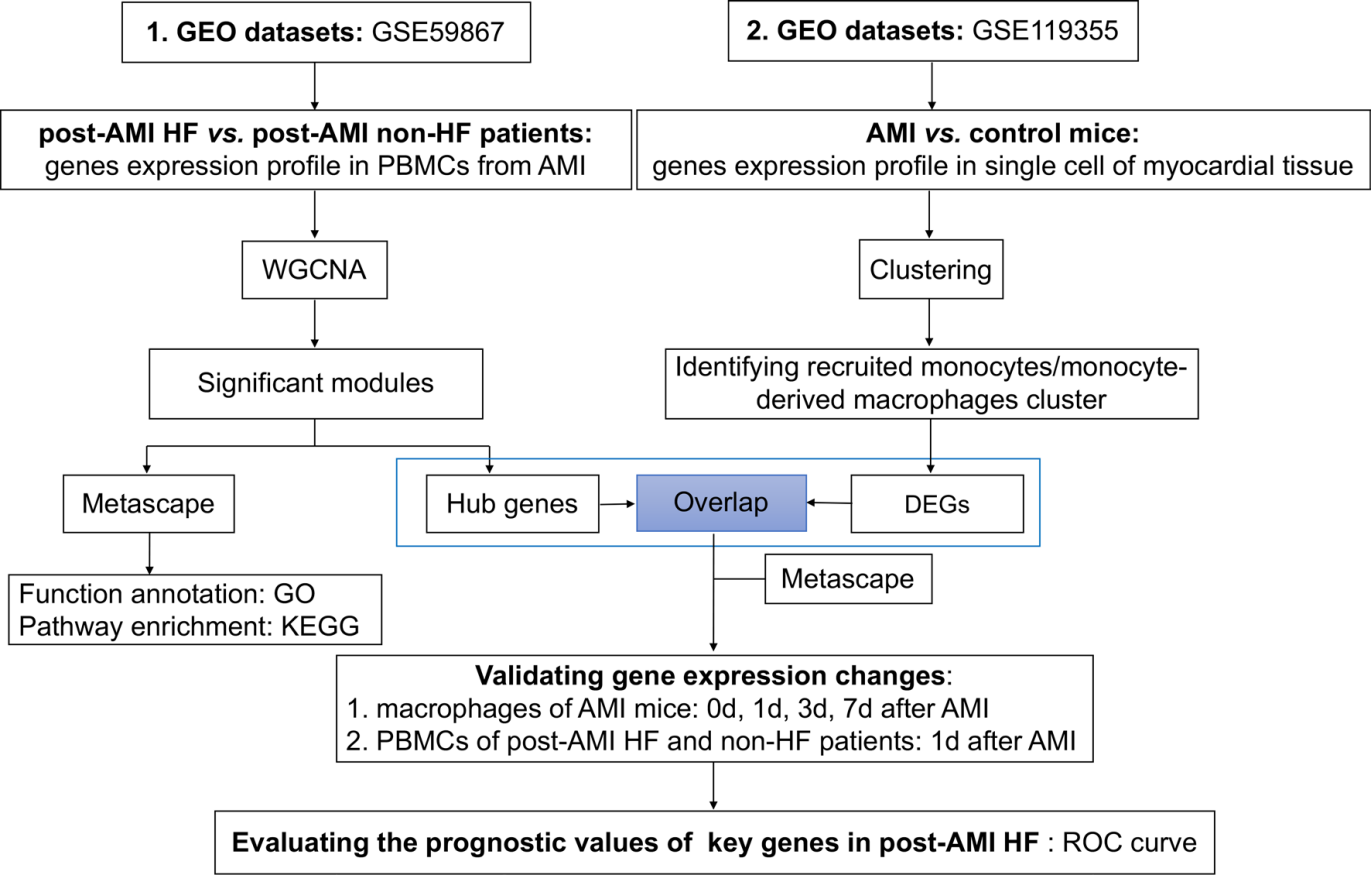
Study flow chart. *GEO* Gene Expression Omnibus, *AMI* acute myocardial infarction, *HF* Heart Failure, *PBMCs* peripheral blood mononuclear cells, *WGCNA* Weighted Gene Co-expression Network Analysis, *GO* Gene Ontology, *KEGG* Kyoto Encyclopedia of Genes and Genomes, *DEGs* differentially expressed genes, *ROC* receiver operating characteristic

The workflow of this study was shown in Fig. 1. The microarray gene expression data of PBMCs and scRNA-seq data of cardiac macrophages were obtained from the Gene Expression Omnibus (GEO) database. The dataset GSE59867 from the Affymetrix Human Gene 1.0 ST Array [transcript (gene) version] [16], dataset GSE119355 from the 10 × Genomics cell ranger platform [25] and published bulk RNA-seq data [26] were utilized in this study. In dataset GSE59867, the data of 17 PBMCs samples from AMI patients (within 1d after infarction) with or without HF development during a period of 6-month follow-up were analyzed (post-AMI HF, n = 9 vs. post-AMI non-HF, n = 8). The gene expression profiling was processed for background correction, log_2_ transformation and quantile normalization using the Robust Multiarray Average (RMA) algorithm. The array probes were mapped with respective gene symbol using the array annotations, and probes lacking annotation information were removed. Genes detected by more than one probe were counted only once. A total of 20,442 genes were included in the following analysis. For dataset GSE119355, the scRNA-seq data of 1806 cardiac macrophages from sham mice and 4697 macrophages from the ischemic area of myocardial infarction were processed using the Cell Ranger Single Cell software suite 1.3.1 by 10 × Genomics (http://10xgenomics.com/). In the validation data, cardiac macrophages of non-infarcted (0 d) or infarcted mice for 1d, 3d, and 7d, isolated from 81 hearts including four biological replicates of 1.5 × 10^6^ cells for each group, were analyzed using bulk RNA-seq. Then the whole transcriptome data were normalized using the method of fragments per kilobase of transcript per million mapped reads (FPKM).

### Weighted gene co-expression network analysis

The ‘WGCNA’ R package was applied to identify disease-related modules and hub genes in these modules [19]. The 20,442 genes in dataset GSE59867 were filtered by median absolute deviation (MAD) to reduce noisy data. The top 10,000 highly variable genes were extracted for network analysis. The soft-threshold method of the Pearson correlation analysis was used to evaluate the possibility that two transcripts construct a weighted network. The appropriate soft-threshold power was screened based on the scale-free topology fit index of 0.8, which was used to calculate the gene co-expression adjacency. The adjacency was transformed into Topological Overlap Matrix (TOM) and the corresponding dissimilarity of genes co-expression (1-TOM) was calculated. Hierarchical clustering of the genes was conducted based on 1-TOM and shown with a clustering tree (dendrogram). Module identification was performed using the Dynamic Tree Cut method with minimal module size of 25 and module detection sensitivity (deepSplit) of 0.5. The cut-off height of 0.3, corresponding to correlation of 0.7 between the modules eigengenes, was chose to merge the similar modules. The correlation between module eigengenes (representing module) and post-AMI HF was calculated, and the modules with correlation coefficient > 0.5 and *p* value < 0.05 were defined to be significantly related to post-AMI HF. Hub genes of each significant module were considered as those with gene significance (|GS|) > 0.35 and module membership (|MM|) > 0.8, showing a significant correlation with the disease trait [27].

### scRNA-seq analysis

The control and post-AMI samples were merged for the following analyses. The standard processing procedures of the dataset GSE119355, including filtering, highly variable genes identification, dimensionality reduction and clustering, were performed using the Scanpy [28]. The low-quality cells expressing < 200 genes and genes expressed in < 3 cells were filtered. To exclude the possible doublets, cells with > 5200 genes were discarded. The poor-quality cells containing > 10% mitochondrial genes were also removed. Based on these criteria, 15,523 genes across 6357 cells in total remained for the subsequent analysis. The gene expression data underwent library-size normalization and log transformation. The highly variable genes with mean expression values between 0.0125 and 1.5 as well as minimal dispersion values of 0.5 were selected. Principal component analysis (PCA) was conducted on the highly variable genes for dimensionality reduction and 18 significant principal components were identified. Clustering in PCA space was performed using a graph-based clustering approach with a resolution of 0.5. The Louvain algorithm was applied for grouping cells into different clusters. Uniform Manifold Approximation and Projection (UMAP) was used for the two-dimensional visualization of the clustering results. The gene expression profile of recruited monocytes/monocytes-derived macrophages cluster (446 cells) were extracted, and DEGs between control and post-AMI group were analyzed by Wilcoxon test. Genes with log_2_^FC^ (fold change, FC, > 1.5) > 0.585 and adjusted p-value (pvals_adj) < 0.05 were considered as significantly DEGs. The top 10 DEGs in each group were visualized using a heatmap.

### Functional enrichment analysis

The genes of the significant modules were used for functional enrichment analysis. The Gene Ontology (GO) biological processes enrichment and Kyoto Encyclopedia of Genes and Genomes (KEGG) pathway analysis were performed via the Metascape [29]. *p* value < 0.05 in GO terms and KEGG pathways were considered statistically significant.

### Statistical analysis

GraphPad Prism 8 was used for analyzing the gene expression level in bulk RNA-seq or microarray dataset. Data was shown as the mean ± standard error of the mean (SEM). The normality of the data was verified by applying Shapiro–Wilk (S–W) normality test. Then the differences between two groups were compared via Mann–Whitney U test for non-normally distributed data or unpaired two-tailed t-test for normally distributed data. Medcalc v19.1 was used for the receiver operator characteristic (ROC) curves analysis. The area under the ROC curve (AUC) was calculated to evaluate the specificity and sensitivity of single gene and their combination via binary logistic regression analysis. For all analyses, *p* value < 0.05 was considered statistically significant.

## Results

### Construction of gene co-expression network and identification of modules

Seventeen PBMCs samples of AMI patients on day 1 after infarction containing 20,442 genes were included, and the top 10,000 highly variable genes were used for the co-expression network construction. When the scale-free fit index reached 0.8, the lowest soft-thresholding power 22 was selected to generate hierarchical clustering of the 10,000 genes (Additional file 1: Figure S1A).

The hierarchical clustering of the genes was analyzed based on 1-TOM. The identified 15 modules and the correlations between genes expression in each module and post-AMI HF were displayed in Additional file 1: Figure S1B (Additional file 5: Table S3) (red, positive correlation; blue, negative correlation). The gray module (insignificant module) containing unassigned genes was discarded.

The co-expression similarity of the 15 modules was quantified by calculating the correlations between module eigengenes. The clustering dendrogram showed that the 15 modules were divided into two main clusters (Additional file 2: Figure S2A), which was also confirmed by the heatmap of module eigengenes adjacencies (Additional file 2: Figure S2B).

### Identification of modules significantly correlated with post-AMI HF and corresponding hub genes

**Fig. 2:**
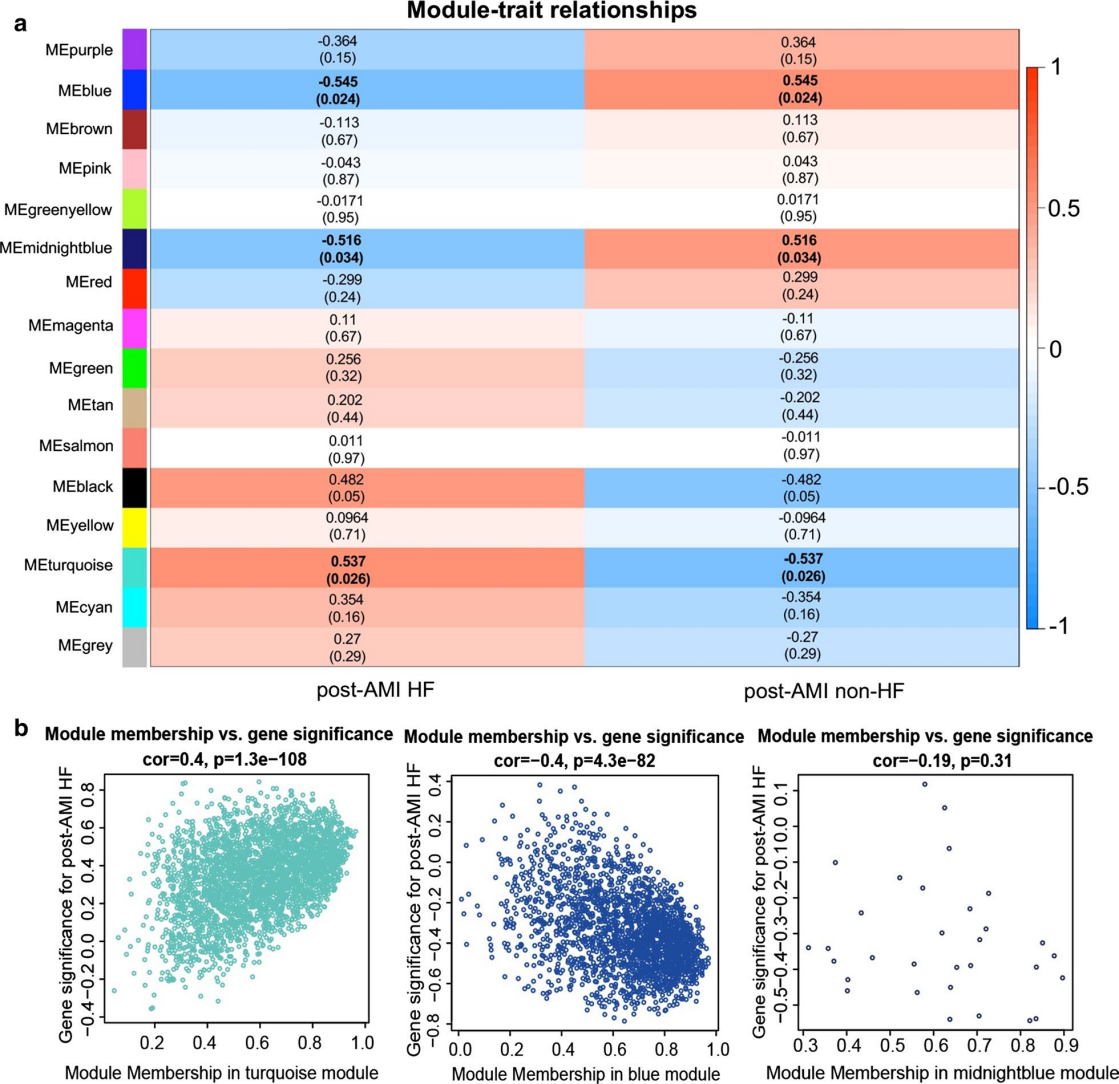
Identification of significant modules highly correlated with post-AMI HF. **a** Heatmap of the correlation between module eigengenes and post-AMI HF. Each row corresponds to a module eigengenes, column to post-AMI HF or non-HF. The correlation coefficient (cor) and p value are shown in each cell. The modules with |cor|> 0.5 and *p* < 0.05 were considered as significantly associated with post-AMI HF. Red, positive correlation; blue, negative correlation. **b** Scatterplots of genes in turquoise, blue and midnightblue modules using the GS and MM measures. Genes in turquoise and blue modules had a high significance for post-AMI HF and high module membership

The results of module-trait relationship analysis showed that 3 of the 15 modules, turquoise, blue and midnightblue module were significantly correlated with post-AMI HF (Fig. 2a). Specifically, the turquoise module was positively associated with post-AMI HF (correlation coefficient = 0.537, *p* < 0.05), whereas the blue (correlation coefficient = − 0.545, *p* < 0.05) and midnightblue modules (correlation coefficient = − 0.516, *p* < 0.05) were negatively associated with the disease. The turquoise, blue and midnightblue modules separately contained 2814, 2115 and 31 genes (Additional file 6: Table S4).

The log_10_ transformation of p value in the linear regression between gene expression and post-AMI HF was used to represent GS. MM was determined as the average absolute GS for all genes in a module. Here, the GS and MM in the turquoise, blue and midnightblue modules were shown as scatterplots (Fig. 2b). Highly significant correlations between GS and MM were only observed in turquoise (correlation coefficient = 0.4, *p* < 0.05) and blue modules (correlation coefficient = -0.4, *p* < 0.05). Based on the cut-off criteria of |MM|> 0.8 and |GS|> 0.35, 382 and 445 highly connected hub genes separately corresponding to the turquoise and blue modules were identified (Additional file 7: Table S5).

### Functional enrichment analysis of genes in significant modules

**Fig. 3:**
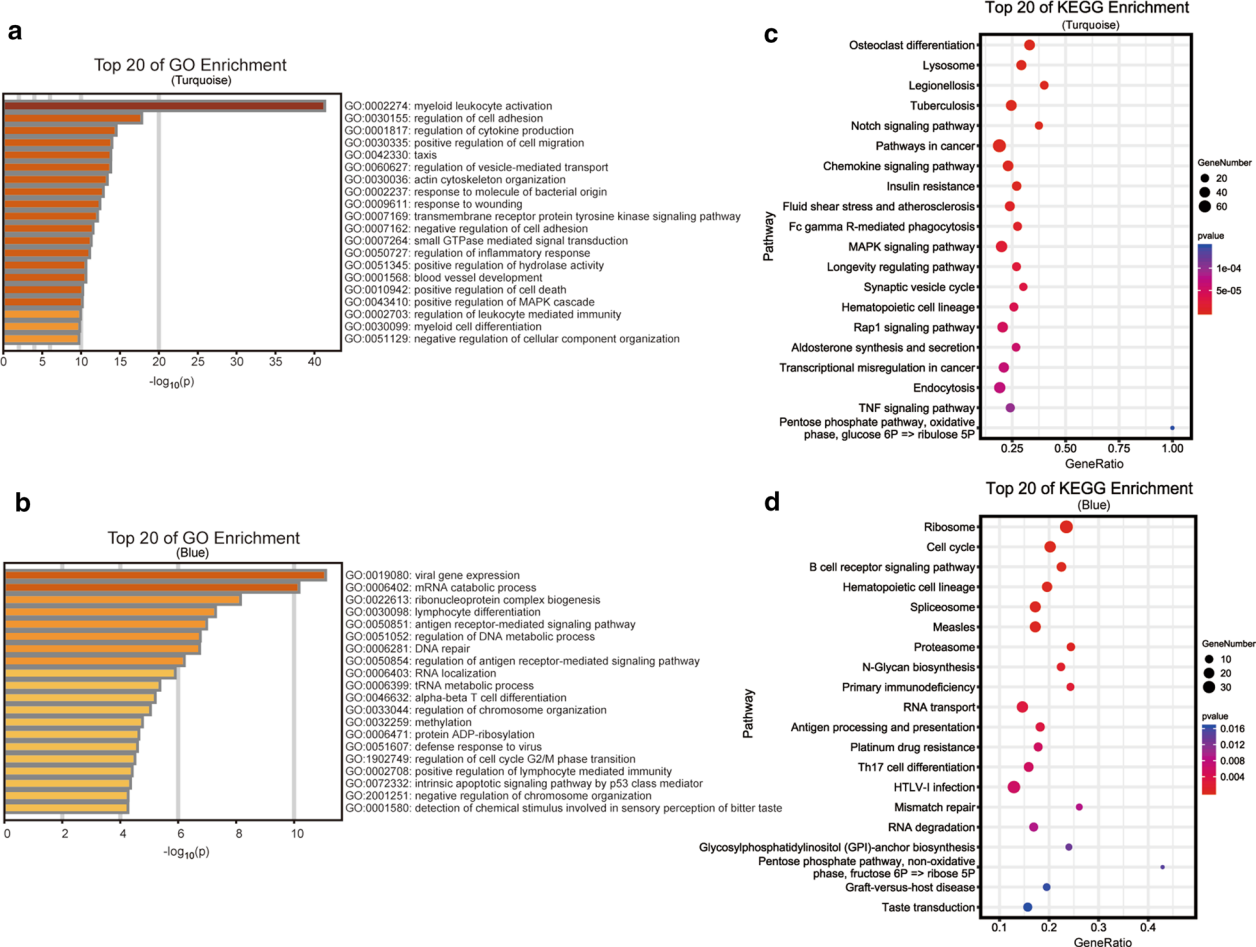
GO enrichment and KEGG pathway analysis of genes in significant modules through Metascape. **a**, **b** Bar chart of GO biological process enrichment of genes in turquoise and blue modules. Top 20 GO terms were shown. The color of the bar represents − log_10_ transformation of *p* value. Gene Ontology: GO; KEGG: Kyoto Encyclopedia of Genes and Genomes. (C-D) Dot plot of KEGG pathway analysis of genes in turquoise and blue modules. Top 20 pathways were shown. Dot size represents the percentage of genes in each pathway. The color scale reflects represents p value

To understand the function of genes in turquoise and blue modules, GO enrichment and KEGG pathway analysis were performed via the Metascape platform. 2814 genes of turquoise module and 2115 genes of blue module were separately uploaded to the platform. The results of biological process in GO enrichment were exhibited in Fig. 3a, b (Additional file 2: Table S1). Genes in turquoise module were enriched in the AMI-related pathological process, such as myeloid leukocyte activation, regulation of cell adhesion, regulation of cytokine production, positive regulation of cell migration, regulation of inflammatory response, and positive regulation of cell death. Genes in blue module were mainly concentrated in the immunity, apoptosis and cell cycle, such as lymphocyte differentiation, antigen receptor-mediated signaling pathway, intrinsic apoptotic signaling pathway by p53 class mediator, regulation of cell cycle G2/M phase transition. The results of KEGG pathway analysis indicated that genes in turquoise modules were mainly involved in Notch signaling pathway, Chemokine signaling pathway, MAPK signaling pathway, TNF signaling pathway, and genes in blue modules were mainly implicated in Cell cycle, Primary immunodeficiency, Antigen processing and presentation, and Th17 cell differentiation (Fig. 3c, d, Additional file 4: Table S2).

### Analysis of differentially expressed genes of recruited monocytes/macrophages in infarcted cardiac tissue

**Fig. 4:**
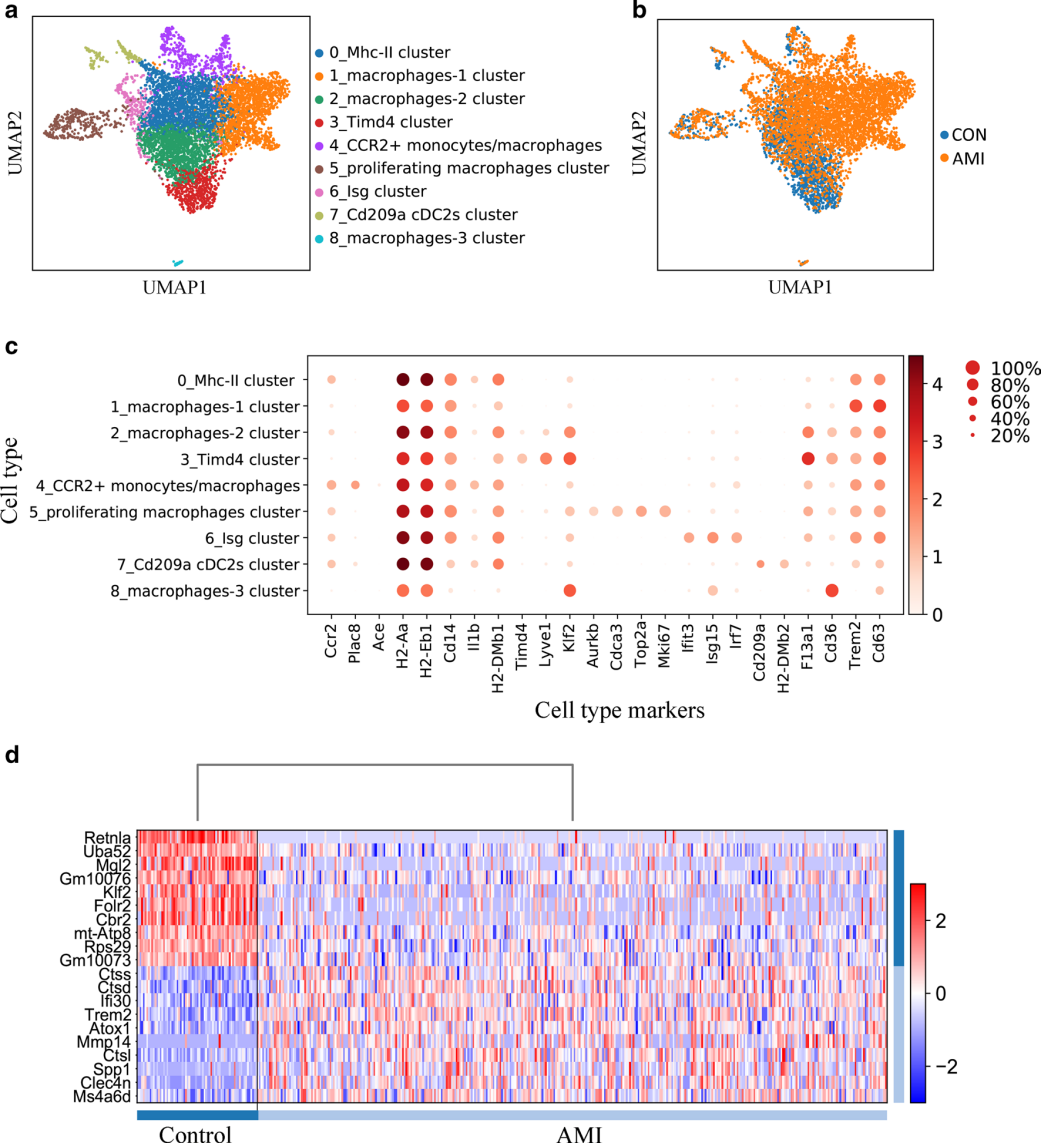
Identifying recruited monocytes/monocytes-derived macrophages cluster (CCR2+ monocytes/macrophages) in the infarcted cardiac tissue of mice. **a** UMAP visualization of cell clusters present in pooled control and post-AMI samples. CCR2+ monocytes/macrophages were identified as cluster 4. **b** UMAP plot depicting all cell types in control (blue) and MI samples (yellow). **c** Dot plot of marker genes for each macrophage cluster. Dot size represents the percentage of cells expressing the marker gene in each cell cluster. The color scale reflects the gene expression level from low to high. **d** Heatmap of top 20 differently expressed genes (10 upregulated and 10 downregulated genes) in CCR2+ monocytes/macrophages between AMI and control mice. The color scale indicates the gene expression level (blue: low; red: high). UMAP: Uniform Manifold Approximation and Projection

In the early stage of AMI, the composition of cardiac macrophages changes dramatically. Circulating pro-inflammatory monocytes (CCR2+) rapidly infiltrate infarcted myocardium within hours, then differentiate into CCR2+ macrophages to promote inflammatory responses, disorders of collagen metabolism, and ultimately contribute to HF pathogenesis [30]. In order to investigate whether some of the hub genes screened above were involved in the regulatory role of recruited monocytes/macrophages in HF development, based on the scRNA-seq data in GSE119355, we identified CCR2+ monocytes/macrophages subset in infarcted cardiac tissue of mice, and obtained the gene expression profiling of each single cell. UMAP analysis for all populations of the cardiac macrophages in both infarcted and non-infarcted mice identified 9 clusters, among which the circulating monocytes derived CCR2+ monocytes/macrophages cluster (macrophages marker: Ccr2, Plac8; monocytes marker: Ace) were focused on this study (Fig. 4a, c). The distribution of all cardiac macrophages from infarcted and non-infarcted mice were shown in Fig. 4b, indicating an obvious increase of macrophages.

Subsequently, we compared the genes expression profiling of CCR2+ monocytes/macrophages cluster in infarcted and non-infarcted mice. Results demonstrated that 103 genes were upregulated, and 278 genes were downregulated in AMI mice compared with controls (Additional file 8: Table S6). The top 20 DEGs (10 upregulated and 10 downregulated) were exhibited in Fig. 4d.

**Fig. 5:**
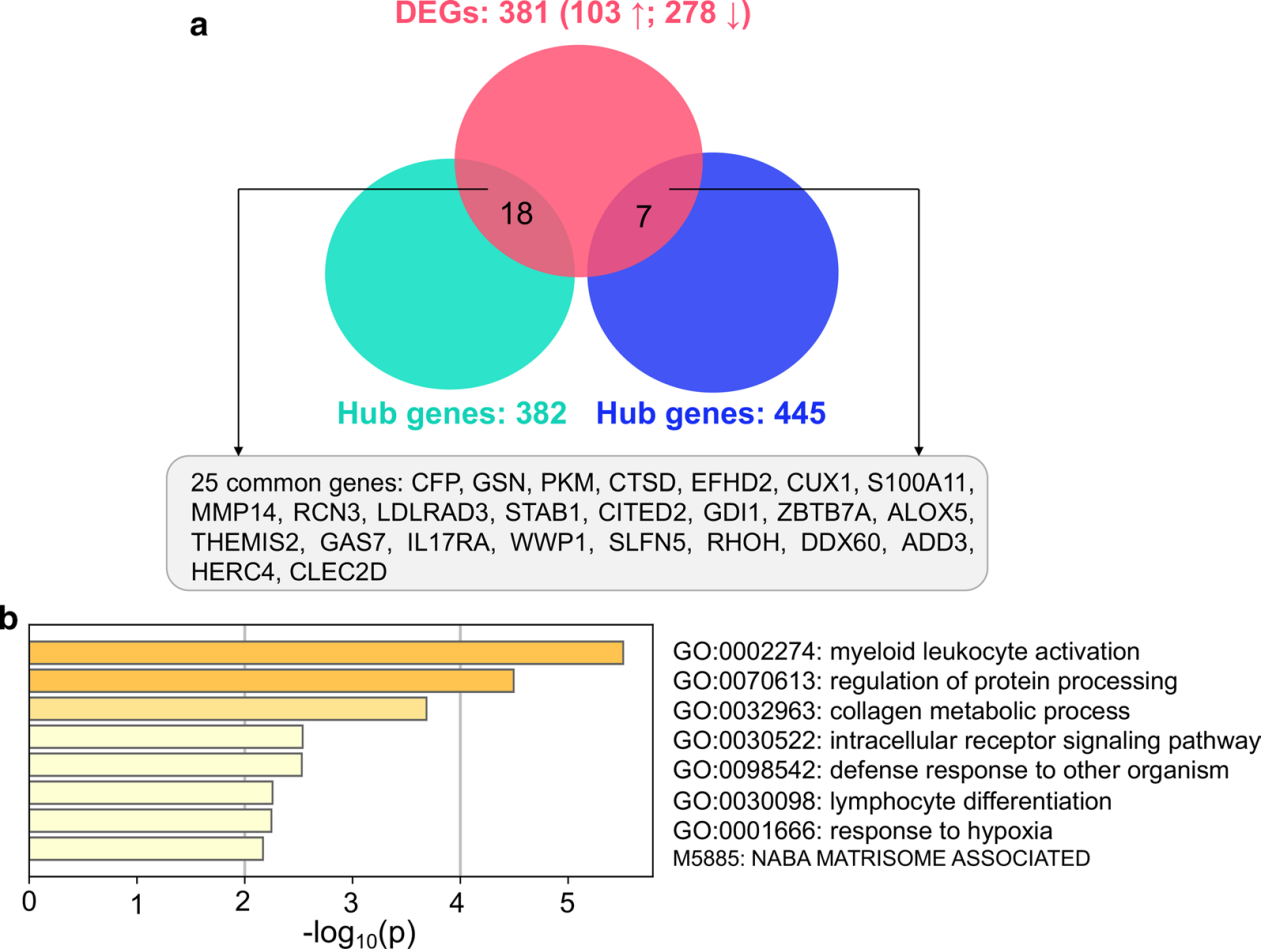
GO biological process enrichment of common genes between DEGs and hub genes. **a** Overlap between DEGs and hub genes of turquoise and blue modules. A total of 25 common genes were identified. **b** The GO biological process enrichment of 25 common genes through Metascape. *DEGs* differentially expressed genes, *GO* Gene Ontology

Furthermore, we found 25 common genes between the 381 DGEs and the 827 hub genes (Fig. 5a). GO biological process enrichment showed that the 25 genes were enriched in myeloid leukocyte activation, collagen metabolic process and response to hypoxia, suggesting a close relationship with cardiac remodeling (Fig. 5b). These 25 genes might be involved in the regulatory role of circulating monocytes in AMI-triggered HF and were selected for the subsequent validation analysis.

### Validation of expression levels of common genes in cardiac macrophages from AMI mice and PBMCs from AMI patients

**Fig. 6:**
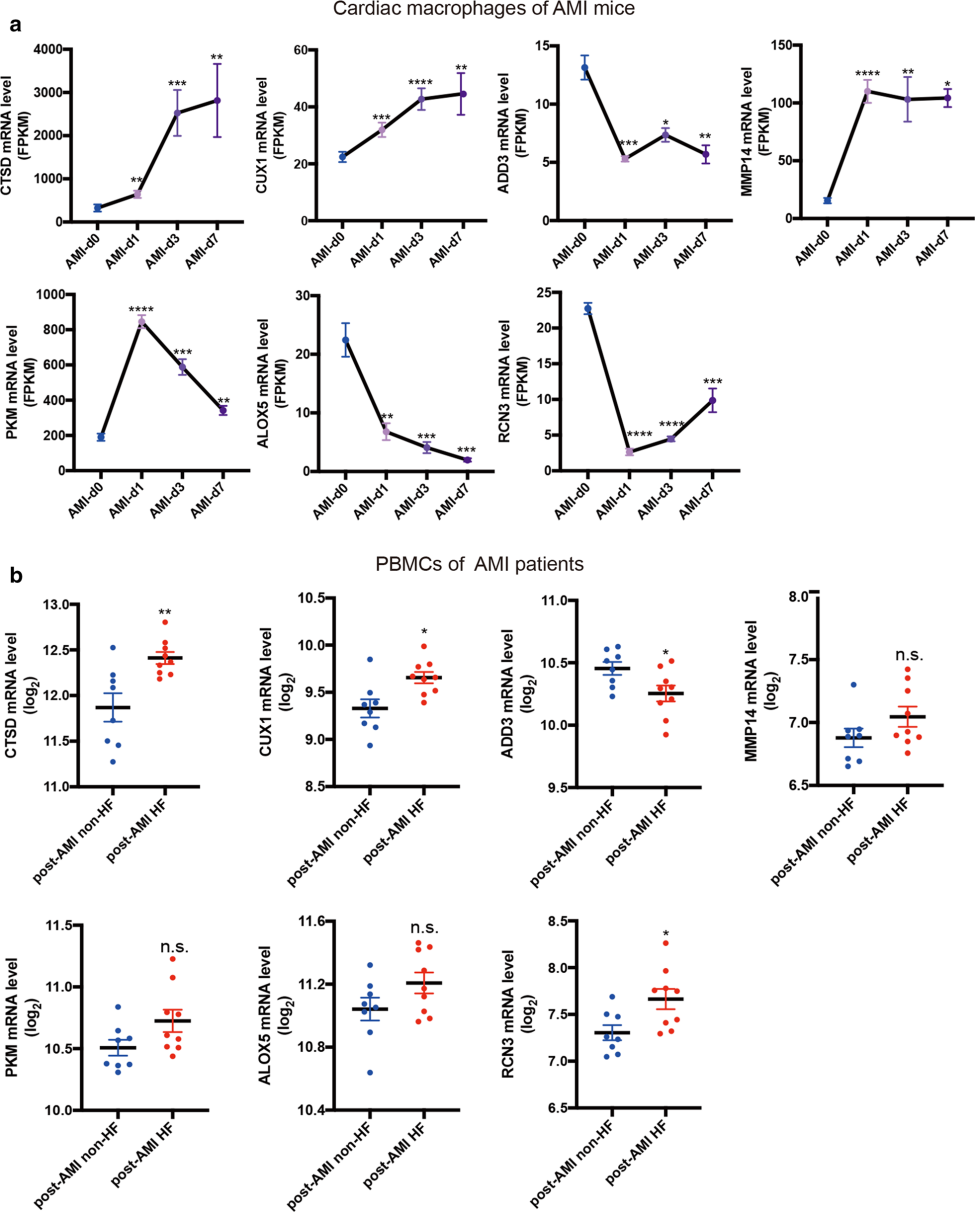
Validation of common genes expression in cardiac macrophages from AMI mice and PBMC from AMI patients. **a** The gene expression changes in cardiac macrophages over the AMI time course, before (day 0) and on days 1, 3 and 7 post AMI, detected by RNA-seq. Seven out of 25 common genes exhibited consistent changes with that in the recruited monocytes/macrophages from infarcted myocardium were shown. *p < 0.05, **p < 0.01 compared with group AMI-d0. **b** The expression levels of 7 common genes in PBMC from AMI patients at admission measured by microarray. *p < 0.05; **p < 0.01 compared with group post-AMI non-HF; n.s.: no significance. post-AMI non-HF group: n = 8, post-AMI HF group: n = 9. Data are presented as mean ± SEM

To determine the reliable biomarkers for the prediction of post-AMI HF, the expression levels of 25 common genes were firstly validated in the bulk RNA-seq data of cardiac macrophages from mice before and on days 1, 3 and 7 post AMI [26]. We observed that 7 out of 25 genes showed the consistent changes with those in the scRNA-seq data, including 4 upregulated genes (MMP14, CUX1, CTSD, PKM) and 3 downregulated genes (ADD3, ALOX5, RCN3) (Fig. 6a). Additionally, the expression levels of the 7 genes were verified in the microarray data of GSE59867 which contained 9 PBMCs samples from AMI patients with HF development and 8 with no HF development. The results showed consistent increase in levels of CUX1 and CTSD and consistent decrease in levels of ADD3 (Fig. 6b). Thus, CUX1, CTSD and ADD3 were ultimately selected for the following ROC curves analysis.

### Effectiveness evaluation of validated genes as prognostic biomarkers of AMI with risk of HF

**Fig. 7:**
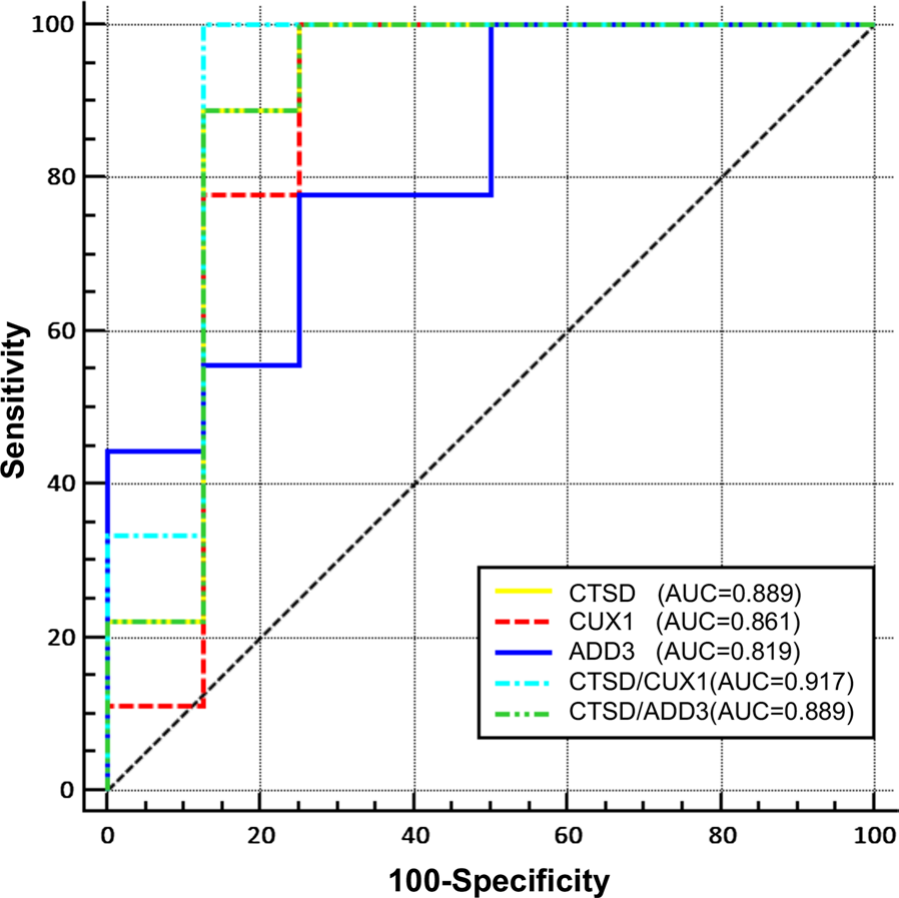
Receiver operator characteristic (ROC) curves analysis of potential biomarkers. The areas under the ROC curves (AUCs) were given for single gene (CUX1, CTSD and ADD3) and combinations (CTSD/CUX1, CTSD/ADD3) to recognize AMI patients who developed HF during a 6-month followed-up. post-AMI non-HF group: n = 8, post-AMI HF group: n = 9. ROC curves were constructed using the log_2_ transformed expression data of GSE59867

To evaluate the power of CUX1, CTSD and ADD3 as predictive biomarkers of HF after AMI, ROC curves analysis was constructed and the AUCs were calculated based on the dataset GSE59867 mentioned above. The corresponding AUCs of CTSD, CUX1 and ADD3 were 0.889 (95% CI 0.644–0.987; *p* = 0.0001), 0.861 (95% CI 0.610–0.960; *p* = 0.0015) and 0.819 (95% CI 0.0.561–0.960; *p* = 0.0023), respectively (Fig. 7, Table 1). The combinations of CTSD and CUX1 had the highest AUC (0.917; CI 0.680–0.995, *p* < 0.0001) (Fig. 7, Table 1), showing the best specificity and sensitivity in identifying AMI patients at risk of HF progression.

**Table 1:** Receiver operating characteristic curves

	AUC	95% CI	*p* value	Cut-off value	Specificity (%)	Sensitivity (%)
CTSD	0.889	0.644–0.987	0.0001	> 12.2266	87.50	88.89
CUX1	0.861	0.610–0.960	0.0015	> 9.3887	75.00	100.00
ADD3	0.819	0.561–0.960	0.0023	≤ 10.3521	75.00	77.78
CTSD/CUX1	0.917	0.680–0.995	< 0.0001	> 0.0102	87.50	100.00
CTSD/ADD3	0.889	0.644–0.987	< 0.0001	> 1.1740	87.50	88.89

*AUC* area under the curve, *CI* confidence interval

## Discussion

In the present study, based upon the gene expression profiling of PBMCs from patients suffered STEMI for 1 day who developed HF or did not during a 6-month follow-up, we identified modules significantly correlated with post-AMI HF as well as hub genes in these modules using WGCNA. Subsequently, we obtained the DEGs of recruited monocytes/macrophages cluster in infarct area of AMI mice. The expression levels of common genes between the hub genes and DEGs were further validated both in cardiac macrophages of another independent group of AMI mice and in PBMCs of AMI patients mentioned above. Consistently upregulated CUX1, CTSD and downregulated ADD3 in these 3 independent studies were identified, and were proved to be potential biomarkers for the early prediction of post-AMI HF. Moreover, the 3 genes might be involved in the regulation of recruited monocytes/macrophages in cardiac remodeling after AMI, and hence serve as potential therapeutic targets of post-AMI HF.

The primarily pathological characteristics of cardiac remodeling during HF development after AMI include inflammatory and immune response, oxidative stress, mitochondrial dysfunction, apoptosis, cardiomyocyte hypertrophy, degradation of the extracellular matrix and fibrosis [8, 9]. In line with these characteristics, functional enrichment analysis in our study showed that genes in HF-related modules mainly participated in inflammatory/inflammation-associated response, immune response, apoptosis and cell cycle transition. The corresponding pathways were Notch signaling pathway, chemokine signaling pathway, MAPK signaling pathway, TNF signaling pathway, cell cycle, antigen processing and presentation, Th17 cell differentiation, DNA replication, all of which play an importantly regulatory role in the cardiac remodeling and consequent HF [8, 9, 31, 32]. These results suggested that AMI patients with developing HF might have more serious inflammation, immunity and apoptosis in infarct zones than whom without HF.

Recruited monocytes/monocyte-derived macrophages in myocardium are key regulators in the AMI-induced cardiac remodeling. The recruited macrophages in infracted myocardium were usually classified into pro-inflammatory (1–4 day post AMI) and anti-inflammatory phenotype (5 days post AMI) [33]. However, growing evidence has indicated that the canonical M1/M2 phenotypes of monocyte-derived macrophages can no longer present all the macrophages subpopulations in infarcted myocardium [34–36]. Defining macrophages only with M1/M2 subtypes may produce misleading conclusions [36]. The high-throughput scRNA-seq can identify unbiased clustering of cells based on the transcriptome analysis at single-cell level, which overcomes the drawback of simple definition of macrophage phenotypes. Therefore, to accurately clarify the relationship between circulating monocytes/monocyte-derived macrophages and post-AMI HF, we obtained the gene expression profiling of the recruited monocytes/macrophages cluster based on the scRNA-seq data of cardiac macrophages from AMI and control mice [25]. Among the 381 DEGs in recruited monocytes/macrophages of infarcted mice, 25 genes overlapped with the hub genes in PBMCs from AMI patients. The functional enrichment analysis of the 25 common genes manifested that they were involved in proinflammatory leukocyte activation, hypoxia response and collagen metabolic process. These results collectively demonstrated that recruited monocytes/macrophages may modulate cardiac remodeling and HF after AMI through these altered genes.

To further determine the reliability of candidate genes as biomarkers for the early recognition of AMI patients at risk of HF, we verified the expression levels of the 25 common genes in cardiac macrophages of mice before and after AMI for 1d, 3d and 7d. There were 7 consistently changed genes in scRNA-seq dataset. There was an overlapping period of recruited macrophages exhibiting pro-inflammatory and anti-inflammatory activity during the first 7 days post AMI [35], which offered an explanation of why each of these 7 genes kept the consistent changes at the different phases of AMI. Among the 7 genes validated in PBMCs from patients at 1 day of AMI (GSE59867), CUX1, CTSD and ADD3 had the similar expression changes to those in the recruited macrophages of AMI mice. A recent study revealed that transcription factor CUX1, a known tumor suppressor [37], is a key regulator of inflammation in rheumatoid arthritis. CUX1 coupled with IκBζ mediated neutrophil and monocyte recruitment by increasing the production of multiple chemokines and cytokines [38], suggesting an adverse effect of CUX1 on cardiac remodeling after AMI. CTSD, a major lysosomal protease, is increasingly known for its involvement in inflammatory response, and has been proposed as biomarkers of several inflammatory diseases, such as non-alcoholic steatohepatitis [39], atherosclerosis and coronary heart disease [40]. CTSD might contribute to plaque vulnerability by inducing macrophages apoptosis [41] and high levels of plasma CTSD was associated with increased risk of future coronary syndromes [42]. Similarly, in dataset GSE59867, CTSD mRNA level in PBMC of post-AMI HF patients at different time points after AMI was higher than that in post-AMI non-HF patients (data not shown). However, the results were inconsistent with that in another study, which indicated that serum CTSD activity decreased in AMI patients with new-onset HF during 6-month follow-up compared to patients without post-AMI HF [43]. Hence, larger clinical trial based on uniform specimen source (PBMCs, plasma or serum) and detecting item (mRNA, protein or protein activity) are necessary to clarify the real relationship between CTSD and post-AMI HF. ADD3, a structural cytoskeletal protein [44], has been implicated to participate in the monocytes migration as well as monocyte-to-macrophage differentiation [45]. The genetic variation of ADD3 was also reported to be associated with left ventricular diastolic relaxation [46]. These findings implied the potential modulation of ADD3 in HF development after AMI.

In the ROC curves analysis, an AUC of 0.7 to 0.8 is regarded acceptable, 0.8 to 0.9 is excellent, and more than 0.9 is outstanding [47]. The AUC values of CUX1, CTSD and ADD3 were all more than 0.8, showing high diagnostic accuracy for identifying AMI patients at risk of HF. In particular, the AUC value of combinations of CTSD and CUX1 reached 0.917, exhibiting outstanding capability to identify the target patients.

## Conclusions

In this study, we constructed the gene co-expression network of PBMCs from AMI patients who developed HF or did not during a 6-month follow-up using WGCNA, and identified significant modules correlated with post-AMI HF. Three hub genes of the significant modules CUX1, CTSD and ADD3 showed the consistent expression changes in PBMCs of AMI patients and recruited monocytes/macrophages of AMI mice, which may mediate the circulating monocytes-triggered cardiac remodeling and HF development. Monocyte-related CUX1, CTSD and ADD3 are promising biomarkers for early identifying AMI patients at risk of HF and potential therapeutic targets of post-AMI HF. Large clinical trials are needed to further validate the predictive value of the 3 genes as biomarkers of HF development after AMI.

## Supplementary Information

**Additional file 1: Figure S1**. Identification of modules through WGCNA. (A) Analysis of scale-free fit index (left panel) and mean connectivity (right panel) for various soft-thresholding powers. The lowest soft-thresholding power was 22 when the scale-free fit index reached 0.8, (B) Clustering dendrogram of genes, with dissimilarity based on TOM, together with the original module colors (Dynamic Tree Cut) and assigned merged module colors (Module colors), as well as the heatmap of correlations between genes expression and post-AMI HF. Under the condition of soft-thresholding power of 22, minimal module size of 25 and cut height of 0.3, 15 modules were identified. A short vertical line in clustering dendrogram corresponds to a gene and highly co-expressed genes are grouped together. Blue: negative correlation; red: positive correlation.

**Additional file 2: Figure S2**. The clustering dendrogram of modules eigengenes and heatmap of the correlations between modules eigengenes. (A) Hierarchical clustering of module eigengenes. Module eigengenes in each branch of the dendrogram were highly positively correlated. (B) Heatmap of the adjacencies in the modules eigengenes network. The modules eigengenes correlations represent modules similarities. Each column and row correspond to one module eigengenes. Red means high adjacency (positive correlation) of two modules, while blue means low adjacency (negative correlation). Red squares along the diagonal are the meta-module.

**Additional file 3: Table S1**. GO biological processes in co-expression modules.

**Additional file 4: Table S2**. KEGG pathways in co-expression modules.

**Additional file 5: Table S3**. Gene_trait_correlation_p-value.

**Additional file 6: Table S4**. Genes of significant modules.

**Additional file 7: Table S5**. Hub genes of turquoise and blue modules.

**Additional file 8: Table S6**. DEGs of AMI vs. Con.

## Data Availability

All data generated or analyzed during this study are included in this published article and its supplementary Additional files. The datasets analyzed during the current study are available in the NCBI—Gene Expression Omnibus (GEO) public repository, [https://www.ncbi.nlm.nih.gov/geo/query/acc.cgi?acc=gse59867] and [https://www.ncbi.nlm.nih.gov/geo/query/acc.cgi?acc=GSE119355]. GEO accession numbers for microarray gene expression data of PBMCs and scRNA-seq data of cardiac macrophages are GSE59867 and GSE119355, respectively.

## Abbreviations

AMI: Acute myocardial infarction
HF: Heart failure
PBMCs: Peripheral blood mononuclear cells
DEGs: Differentially expressed genes
STEMI: ST-segment-elevation myocardial infarction
NPs: Natriuretic peptides
sST2: Soluble suppression of tumorigenicity-2
GDF-15: Growth/differential factor-15
WGCNA: Weighted gene co-expression network analysis
scRNA-seq: Single-cell RNA-sequencing
Bulk RNA-seq: Bulk RNA-sequencing
GEO: Gene Expression Omnibus
RMA: Robust Multiarray Average
FPKM: Fragments per kilobase of transcript per million mapped reads
MAD: Median absolute deviation
TOM: Topological overlap Matrix
GS: Gene significance
MM: Module membership
cor: Correlation coefficient
PCA: Principal component analysis
UMAP: Uniform Manifold Approximation and Projection
pvals_adj: Adjusted p-value
GO: Gene Ontology
KEGG: Kyoto Encyclopedia of Genes and Genomes
SEM: Standard error of the mean
ROC: Receiver operator characteristic curves
AUC: Area under the ROC curve
CCR2+: Circulating pro-inflammatory monocytes
