# Supplementary figures and images for: Identification of monocyte-associated genes as predictive biomarkers of heart failure after acute myocardial infarction
Source: BMC Med Genomics. 2021 Feb 9;14:44. doi: 10.1186/s12920-021-00890-6 (PMC7871627; doi:10.1186/s12920-021-00890-6)

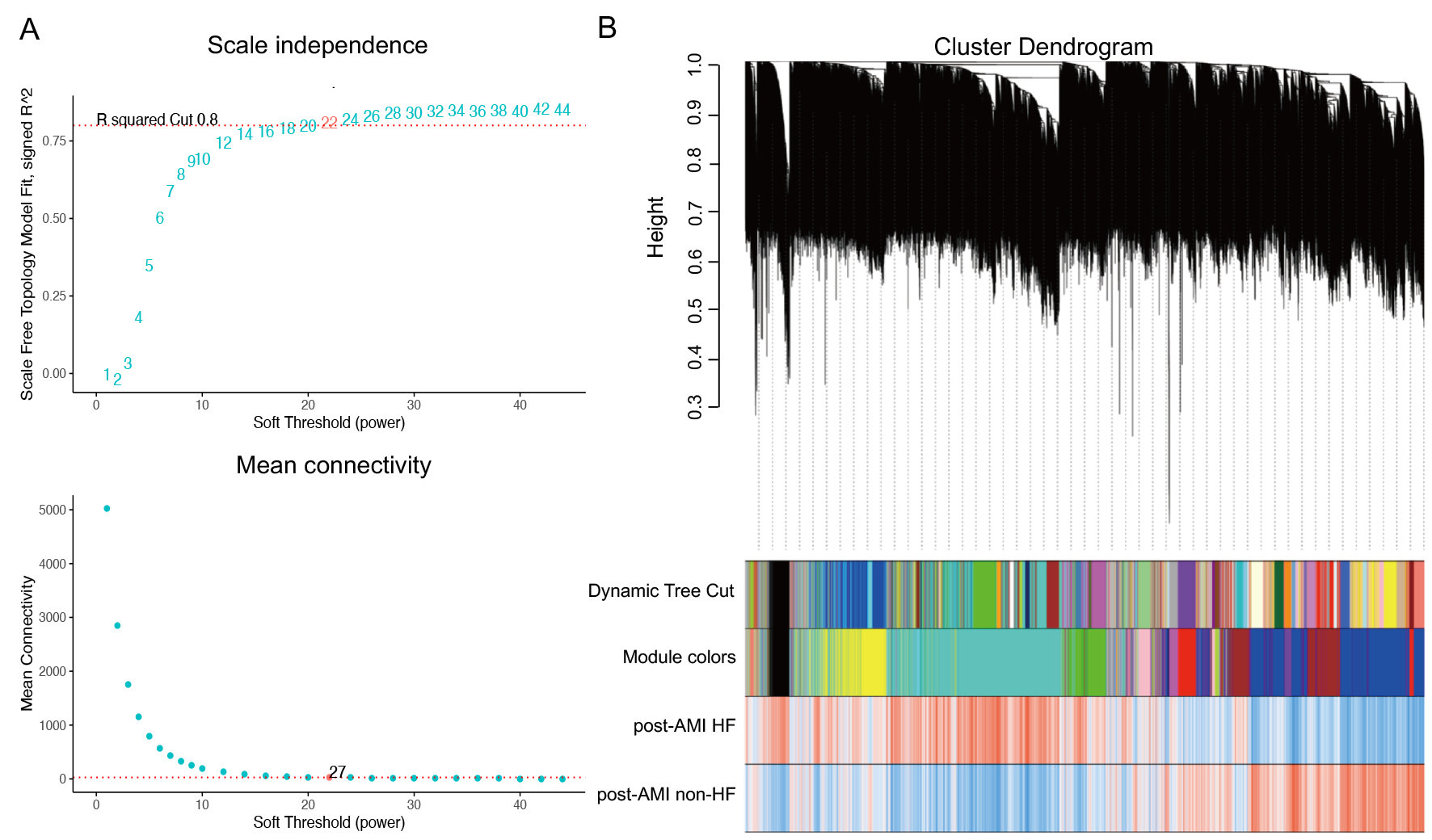

Supplement: Supplementary file 1 — Additional file 1: Figure S1. Identification of modules through WGCNA. (A) Analysis of scale-free fit index (left panel) and mean connectivity (right panel) for various soft-thresholding powers. The lowest soft-thresholding power was 22 when the scale-free fit index reached 0.8, (B) Clustering dendrogram of genes, with dissimilarity based on TOM, together with the original module colors (Dynamic Tree Cut) and assigned merged module colors (Module colors), as well as the heatmap of correlations between genes expression and post-AMI HF. Under the condition of soft-thresholding power of 22, minimal module size of 25 and cut height of 0.3, 15 modules were identified. A short vertical line in clustering dendrogram corresponds to a gene and highly co-expressed genes are grouped together. Blue: negative correlation; red: positive correlation. [file 12920_2021_890_MOESM1_ESM.tif]

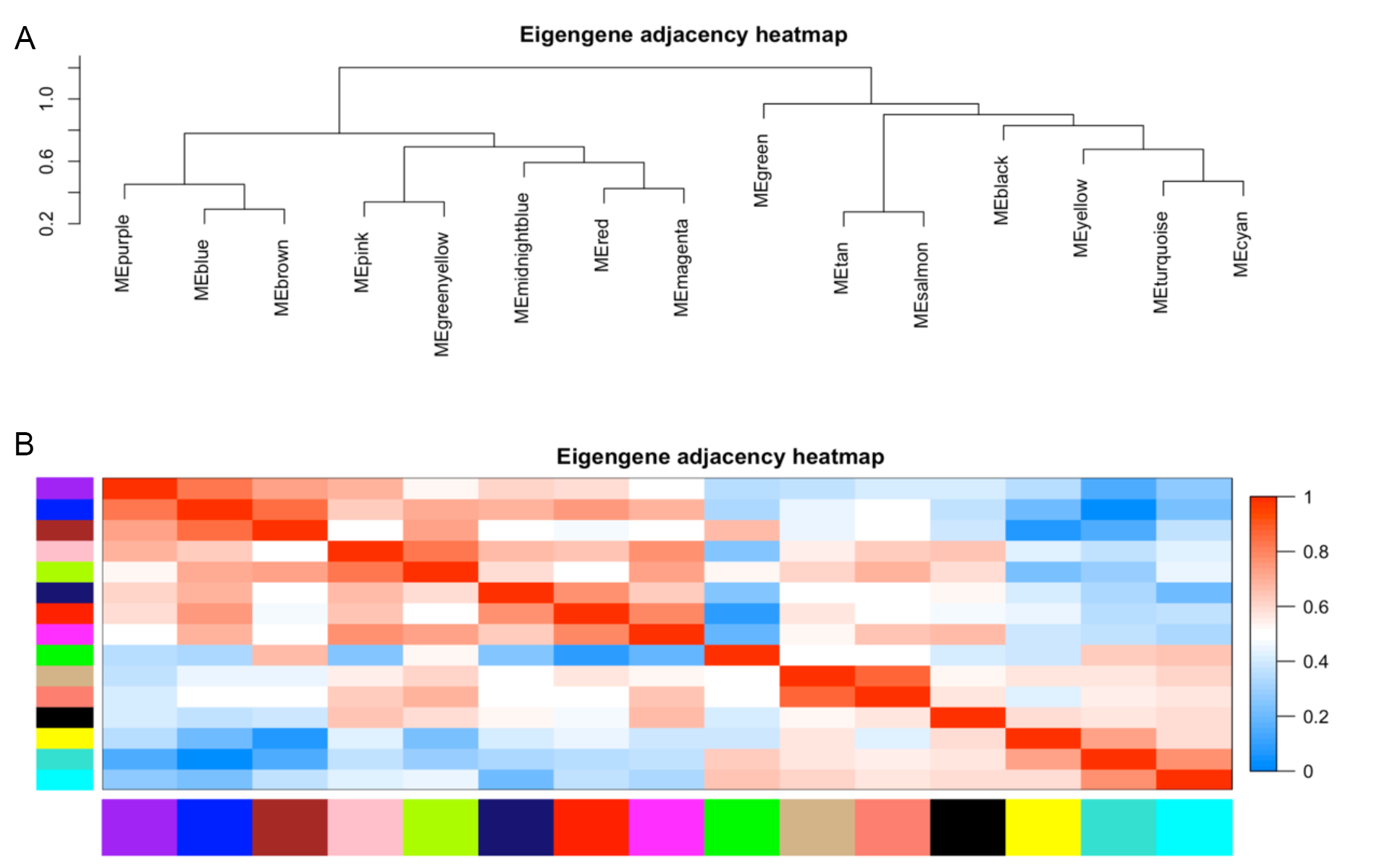

Supplement: Supplementary file 2 — Additional file 2: Figure S2. The clustering dendrogram of modules eigengenes and heatmap of the correlations between modules eigengenes. (A) Hierarchical clustering of module eigengenes. Module eigengenes in each branch of the dendrogram were highly positively correlated. (B) Heatmap of the adjacencies in the modules eigengenes network. The modules eigengenes correlations represent modules similarities. Each column and row correspond to one module eigengenes. Red means high adjacency (positive correlation) of two modules, while blue means low adjacency (negative correlation). Red squares along the diagonal are the meta-module. [file 12920_2021_890_MOESM2_ESM.tif]
