# Supplementary material for: Identification of monocyte-associated genes as predictive biomarkers of heart failure after acute myocardial infarction
Source: BMC Med Genomics. 2021 Feb 9;14:44. doi: 10.1186/s12920-021-00890-6 (PMC7871627; doi:10.1186/s12920-021-00890-6)
Supplement: Supplementary file 3 — Additional file 3: Table S1. GO biological processes in co-expression modules. [file 12920_2021_890_MOESM3_ESM.docx]

**TABLE S1. GO biological processes in co-expression modules**

| **Term** | **Description** | **Gene counts** | **Percent** | **Log p** | **Log(q-value)** |
| --- | --- | --- | --- | --- | --- |
| **Turquoise** |  |  |  |  |  |
| GO:0002274 | myeloid leukocyte activation | 199 | 0.30 | -41.49 | -37.29 |
| GO:0030155 | regulation of cell adhesion | 160 | 0.22 | -17.88 | -14.80 |
| GO:0001817 | regulation of cytokine production | 157 | 0.21 | -14.59 | -11.54 |
| GO:0030335 | positive regulation of cell migration | 125 | 0.22 | -14.04 | -11.04 |
| GO:0042330 | taxis | 139 | 0.21 | -13.91 | -10.94 |
| GO:0060627 | regulation of vesicle-mediated transport | 122 | 0.22 | -13.87 | -10.94 |
| GO:0030036 | actin cytoskeleton organization | 144 | 0.21 | -13.45 | -10.57 |
| GO:0002237 | response to molecule of bacterial origin | 89 | 0.25 | -12.92 | -10.15 |
| GO:0009611 | response to wounding | 141 | 0.20 | -12.49 | -9.77 |
| GO:0007169 | transmembrane receptor protein tyrosine kinase signaling pathway | 147 | 0.20 | -12.19 | -9.49 |
| GO:0007162 | negative regulation of cell adhesion | 75 | 0.26 | -11.59 | -8.97 |
| GO:0007264 | small GTPase mediated signal transduction | 117 | 0.21 | -11.36 | -8.76 |
| GO:0050727 | regulation of inflammatory response | 109 | 0.21 | -11.17 | -8.63 |
| GO:0051345 | positive regulation of hydrolase activity | 148 | 0.19 | -10.69 | -8.20 |
| GO:0001568 | blood vessel development | 148 | 0.19 | -10.69 | -8.20 |
| GO:0010942 | positive regulation of cell death | 141 | 0.19 | -10.26 | -7.84 |
| GO:0043410 | positive regulation of MAPK cascade | 113 | 0.20 | -10.21 | -7.80 |
| GO:0002703 | regulation of leukocyte mediated immunity | 56 | 0.27 | -9.96 | -7.59 |
| GO:0030099 | myeloid cell differentiation | 92 | 0.22 | -9.86 | -7.52 |
| GO:0051129 | negative regulation of cellular component organization | 143 | 0.19 | -9.79 | -7.47 |
| **Blue** |  |  |  |  |  |
| GO:0019080 | viral gene expression | 47 | 0.24 | -11.10 | -6.94 |
| GO:0006402 | mRNA catabolic process | 69 | 0.19 | -10.18 | -6.76 |
| GO:0022613 | ribonucleoprotein complex biogenesis | 75 | 0.16 | -8.16 | -5.08 |
| GO:0030098 | lymphocyte differentiation | 60 | 0.17 | -7.30 | -4.37 |
| GO:0050851 | antigen receptor-mediated signaling pathway | 55 | 0.17 | -6.99 | -4.16 |
| GO:0051052 | regulation of DNA metabolic process | 69 | 0.16 | -6.77 | -3.99 |
| GO:0006281 | DNA repair | 82 | 0.15 | -6.75 | -3.98 |
| GO:0050854 | regulation of antigen receptor-mediated signaling pathway | 19 | 0.29 | -6.22 | -3.51 |
| GO:0006403 | RNA localization | 42 | 0.18 | -5.90 | -3.24 |
| GO:0006399 | tRNA metabolic process | 35 | 0.18 | -5.38 | -2.76 |
| GO:0046632 | alpha-beta T cell differentiation | 23 | 0.23 | -5.22 | -2.63 |
| GO:0033044 | regulation of chromosome organization | 54 | 0.15 | -5.05 | -2.50 |
| GO:0032259 | methylation | 54 | 0.15 | -4.77 | -2.25 |
| GO:0006471 | protein ADP-ribosylation | 12 | 0.32 | -4.65 | -2.17 |
| GO:0051607 | defense response to virus | 40 | 0.16 | -4.60 | -2.13 |
| GO:1902749 | regulation of cell cycle G2/M phase transition | 36 | 0.17 | -4.51 | -2.08 |
| GO:0002708 | positive regulation of lymphocyte mediated immunity | 22 | 0.21 | -4.42 | -2.03 |
| GO:0072332 | intrinsic apoptotic signaling pathway by p53 class mediator | 18 | 0.23 | -4.36 | -2.00 |
| GO:2001251 | negative regulation of chromosome organization | 27 | 0.18 | -4.28 | -1.96 |
| GO:0001580 | detection of chemical stimulus involved in sensory perception of bitter taste | 12 | 0.30 | -4.27 | -1.96 |

GO: Gene Ontology.
