# Supplementary material for: Identification of monocyte-associated genes as predictive biomarkers of heart failure after acute myocardial infarction
Source: BMC Med Genomics. 2021 Feb 9;14:44. doi: 10.1186/s12920-021-00890-6 (PMC7871627; doi:10.1186/s12920-021-00890-6)
Supplement: Supplementary file 4 — Additional file 4: Table S2. KEGG pathways in co-expression modules. [file 12920_2021_890_MOESM4_ESM.docx]

**TABLE S2. KEGG pathways in co-expression modules**

| **Term** | **Description** | **Gene counts** | **Percent** | **Log p** | **Log(q-value)** |
| --- | --- | --- | --- | --- | --- |
| **Turquoise** |  |  |  |  |  |
| hsa04380 | Osteoclast differentiation | 43 | 0.33 | -10.78 | -8.09 |
| hsa04142 | Lysosome | 36 | 0.29 | -7.55 | -5.32 |
| hsa05134 | Legionellosis | 22 | 0.40 | -7.53 | -5.32 |
| hsa05152 | Tuberculosis | 44 | 0.25 | -6.60 | -4.50 |
| hsa04330 | Notch signaling pathway | 18 | 0.38 | -5.79 | -3.80 |
| hsa05200 | Pathways in cancer | 75 | 0.19 | -5.68 | -3.77 |
| hsa04062 | Chemokine signaling pathway | 42 | 0.23 | -5.56 | -3.71 |
| hsa04931 | Insulin resistance | 29 | 0.27 | -5.47 | -3.68 |
| hsa05418 | Fluid shear stress and atherosclerosis | 34 | 0.24 | -4.99 | -3.25 |
| hsa04666 | Fc gamma R-mediated phagocytosis | 25 | 0.27 | -4.91 | -3.22 |
| hsa04010 | MAPK signaling pathway | 51 | 0.20 | -4.70 | -3.04 |
| hsa04211 | Longevity regulating pathway | 24 | 0.27 | -4.60 | -2.98 |
| hsa04721 | Synaptic vesicle cycle | 19 | 0.30 | -4.49 | -2.91 |
| hsa04640 | Hematopoietic cell lineage | 25 | 0.26 | -4.40 | -2.86 |
| hsa04015 | Rap1 signaling pathway | 43 | 0.20 | -4.31 | -2.82 |
| hsa04925 | Aldosterone synthesis and secretion | 22 | 0.27 | -4.23 | -2.79 |
| hsa05202 | Transcriptional misregulation in cancer | 38 | 0.21 | -4.18 | -2.76 |
| hsa04144 | Endocytosis | 50 | 0.19 | -4.16 | -2.76 |
| hsa04668 | TNF signaling pathway | 26 | 0.24 | -4.01 | -2.64 |
| M00006 | Pentose phosphate pathway, oxidative phase, glucose 6P => ribulose 5P | 4 | 1.00 | -3.82 | -2.51 |
| **Blue** |  |  |  |  |  |
| hsa03010 | Ribosome | 36 | 0.24 | -8.30 | -5.61 |
| hsa04110 | Cell cycle | 25 | 0.20 | -4.71 | -2.31 |
| hsa04662 | B cell receptor signaling pathway | 16 | 0.23 | -3.81 | -1.60 |
| hsa04640 | Hematopoietic cell lineage | 19 | 0.20 | -3.56 | -1.47 |
| hsa03040 | Spliceosome | 23 | 0.17 | -3.29 | -1.38 |
| hsa05162 | Measles | 23 | 0.17 | -3.29 | -1.38 |
| hsa03050 | Proteasome | 11 | 0.24 | -3.11 | -1.32 |
| hsa00510 | N-Glycan biosynthesis | 11 | 0.22 | -2.78 | -1.04 |
| hsa05340 | Primary immunodeficiency | 9 | 0.24 | -2.62 | -0.93 |
| hsa03013 | RNA transport | 25 | 0.15 | -2.51 | -0.87 |
| hsa04612 | Antigen processing and presentation | 14 | 0.18 | -2.46 | -0.87 |
| hsa01524 | Platinum drug resistance | 13 | 0.18 | -2.24 | -0.76 |
| hsa04659 | Th17 cell differentiation | 17 | 0.16 | -2.23 | -0.76 |
| hsa05166 | HTLV-I infection | 33 | 0.13 | -2.23 | -0.76 |
| hsa03430 | Mismatch repair | 6 | 0.26 | -2.06 | -0.63 |
| hsa03018 | RNA degradation | 13 | 0.17 | -2.04 | -0.63 |
| hsa00563 | Glycosylphosphatidylinositol (GPI)-anchor biosynthesis | 6 | 0.24 | -1.87 | -0.48 |
| M00007 | Pentose phosphate pathway, non-oxidative phase, fructose 6P => ribose 5P | 3 | 0.43 | -1.83 | -0.48 |
| hsa05332 | Graft-versus-host disease | 8 | 0.20 | -1.79 | -0.47 |
| hsa04742 | Taste transduction | 13 | 0.16 | -1.78 | -0.47 |

KEGG: Kyoto Encyclopedia of Genes and Genomes.
